# Acute Ulnar Neuropathy After Uncomplicated Contraceptive Implant Insertion: A Case Report

**DOI:** 10.7759/cureus.28161

**Published:** 2022-08-19

**Authors:** S Ahmed Hussain, Danielle Holland

**Affiliations:** 1 Gynecologic Surgery and Obstetrics, Royal Air Force (RAF) Lakenheath Hospital, Lakenheath, GBR

**Keywords:** contraception, implant complications, ulnar neuropathy, ulnar nerve, contraceptive implant

## Abstract

To avoid the nerves and blood vessels of the upper extremity, the subdermal contraceptive implant Nexplanon (N.V. Organon, Oss, The Netherlands) is inserted into the subdermis at a location 8-10 cm proximal to the medial epicondyle of the humerus and 3-5 cm posterior to the groove between the biceps and triceps muscles. Ulnar nerve injury is a known but extremely rare complication associated with implant insertion. Risk factors for ulnar nerve insult include inadvertent deep insertion, device migration, and an underweight patient. This is a case report of an obese 40-year-old female who presented four days after uncomplicated contraceptive implant insertion with symptoms consistent with an acute ulnar neuropathy, despite the implant being located in the proper superficial location. Symptoms resolved soon after implant removal. This unique presentation may be explained by the aberrant anatomy of the ulnar nerve. It is important for clinicians to be able to recognize and treat this rare complication of contraceptive implant insertion, even in a patient who possesses no risk factors for ulnar nerve injury.

## Introduction

The subdermal contraceptive implant is a popular and reliable method of contraception. The currently available device (Nexplanon®, N.V. Organon, Oss, The Netherlands) consists of a single radio-opaque plastic rod 40 mm in length and 2 mm in diameter that contains 68 mg of etonogestrel, a progestin [1]. The device initially releases 60-70 mcg per day of etonogestrel and decreases to 30 mcg per day after three years, which is the recommended duration of use [2]. The manufacturer reports a failure rate of 0.38 pregnancies per 100 woman-years of use, which makes it the most reliable form of reversible contraception [3].

Insertion is performed in the outpatient setting under local anesthetic. To avoid injury to the major neurovascular structures of the upper extremity, the device is deployed 8-10 cm proximal to the medial epicondyle of the humerus and 3-5 cm posterior to the groove between the triceps and biceps muscles [4]. The implant is then deployed by the single-use insertion device supplied by the manufacturer.

Complications with insertion are uncommon but include infection, hematoma, expulsion, and allergic reaction [5]. Ulnar neuropathy is an extremely rare sequela with an incidence of less than 1% and is more often associated with implant removal rather than insertion [6]. Most instances of insertional insult occur after improperly deep insertion, after device migration, or in underweight women whose thin adipose layer predisposes the nerve to insult [6,7]. There have been no cases reported in the literature of ulnar neuropathy related to contraceptive implant insertion in patients without these risk factors. This presentation describes the first reported case of acute ulnar neuropathy following superficial subdermal insertion of a Nexplanon® contraceptive implant in an obese female.

## Case presentation

A 40-year-old, right-hand-dominant, Caucasian female presented to the Women’s Health Clinic for the removal of her levonorgestrel-containing intrauterine device and subsequent insertion of a Nexplanon® contraceptive implant. The patient reported a personal history of a lower extremity deep vein thrombosis and migraines with aura, limiting hormonal contraception options to those without estrogen. The patient’s medical history was otherwise unremarkable. Vital signs were within the normal range. Her weight was 92 kg, and her BMI was 33 kg/m^2^.

The patient’s intrauterine device was removed. The insertion of the contraceptive implant in the left upper extremity was uncomplicated and was accomplished per the manufacturer’s instructions. Both the patient and the physician palpated the implant in its expected superficial subdermal location, 8 cm proximal to the medial epicondyle of the humerus and 3 cm posterior to the sulcus groove between the triceps and the biceps. The patient had no significant post-insertion pain and was discharged from the clinic in good condition.

The patient returned to the clinic on the fourth day after insertion due to significant left upper extremity pain. On questioning, she reported no issues immediately after implant insertion. However, approximately five hours after insertion, she reported the onset of a “shooting” pain that radiated from the insertion site to the medial epicondyle of the humerus and distally to the fourth and fifth digits. This pain was described as a constant, unbearable “searing” or “burning” sensation that was significantly exacerbated by the elevation of the left upper extremity. She additionally reported allodynia, with significant pain caused by any pressure on the implant location including light brushing from clothing. The pain had continued to worsen since its onset, culminating in her acute return to the clinic.

On physical examination, the patient’s vital signs were within the normal range. The implant insertion site was normal in appearance, without evidence of erythema, ecchymosis, hematoma, induration, or discharge. The implant was palpated in its expected superficial subdermal location, and on manipulation of the implant, its entire outline could be visualized beneath the dermis. Palpation of the implant caused a marked exacerbation of the patient’s pain. Sensation to touch was decreased to 2+ along the left fifth digit and medial aspect of the left fourth digit. The patient was able to move these digits without issue. Grip strength appeared to be normal (5+) on the left hand, although her effort was limited by pain. There were no contralateral sensory or motor deficits.

Considering the clinical presentation, removal of the contraceptive implant was recommended, and the patient consented to the same. Due to the ease of palpation of the device, no imaging was obtained. Direct visualization of the device within the incision confirmed that the implant was in its expected superficial location. Implant removal was accomplished easily. The implant was removed intact. The patient reported immediate improvement, although not complete resolution, of her pain.

On a follow-up telephone encounter seven days later, the patient reported complete resolution of her symptoms and opted for progesterone-only oral pills for contraception.

## Discussion

The ulnar nerve originates from the brachial plexus, containing fibers from the C8-T1 nerve roots. It travels along the medial aspect of the upper extremity. At the midpoint of the arm, it enters the fascia and continues along the medial head of the triceps muscle. It continues distally along the posterior aspect of the medial epicondyle, entering the forearm between the heads of the flexor carpi ulnaris. It then travels deep along the ulna where it separates into its muscular, palmar cutaneous, and dorsal cutaneous branches [8].

The ulnar nerve supplies sensory innervation to the medial half of the fourth digit and the entire fifth digit, and the corresponding palmar and dorsal areas of the hand. It supplies motor innervation to the muscles of the anterior compartment of the forearm and the majority of the intrinsic muscles of the hand [9].

This patient’s painful paresthesias corresponded exactly to the course and area of innervation of the ulnar nerve, and thus, they represent a classic presentation of sensory ulnar neuropathy [6]. Such an ulnar nerve insult is an exceedingly rare complication of contraceptive implant placement, with only three cases reported in the literature [6]. Instead, the majority of such cases occur in patients who have undergone a technically challenging removal of a deeply placed device but who had no symptoms while the implant was in place [6].

For the first time in a female without risk factors for ulnar nerve insult, we report a neuropathy that occurred immediately after the superficial insertion of a contraceptive implant. These risk factors include an underweight patient, improperly deep placement, and migration to a deep location after correct insertion.

The device was placed at the site suggested by the manufacturer and was confirmed to be in its expected superficial subdermal position even at the peak of the patient’s symptoms. This case is especially notable because palpation of the superficial device, whose outline could be easily identified beneath the dermis, resulted in the exacerbation of the patient’s paresthesias despite several centimeters of adipose tissue buffering the device from the expected location of the ulnar nerve.

There are two possible explanations for this patient’s unique clinical course. The first is a normally located ulnar nerve that is extremely sensitive to insult. This is unlikely in this patient given the diminutive height of the contraceptive implant (2 mm) in comparison to the thickness of the adiposity between the implant and the normal location of the ulnar nerve. Additionally, the patient reported that she had never experienced similar paresthesia prior to implant insertion. If there were a preexisting ulnar nerve hypersensitivity, the patient would likely have felt a similar sensation in response to pressure at the medial upper extremity at some point during her lifetime.

A more likely scenario is an aberrant course or branching of the ulnar nerve. There has been one report in the literature of an anatomic variation in which the ulnar nerve produced a branch above the elbow, which continued distally without penetrating the fascia of the muscles of the forearm [10]. The patient’s clinical course could be explained by an insult to an abnormally superficial branch of the ulnar nerve that continues distally to innervate its usual sensory areas. Additionally, a cadaver study found that up to 8% of ulnar nerves have some aberrance, such as abnormal communication with a neighboring nerve [11]. The patient’s symptoms could also be explained by an insult to an unrelated superficial nerve with an abnormal communication to the ulnar nerve.

## Conclusions

Due to the rarity of nerve injuries associated with contraceptive implant insertion, there is a dearth of literature describing their presentation, etiology, and treatment. In this report, we describe a unique occurrence of one such neuropathy in a patient without risk factors and whose symptoms resolved after the removal of the implant.

Prompt removal of the contraceptive implant should be considered the first-line treatment for patients presenting with acute ulnar neuropathy, even when the superficial location of the implant would suggest an ulnar nerve injury is unlikely.
